# The dynamics of COVID-19 in the UAE based on fractional derivative modeling using Riesz wavelets simulation

**DOI:** 10.1186/s13662-021-03262-7

**Published:** 2021-02-19

**Authors:** Mutaz Mohammad, Alexander Trounev, Carlo Cattani

**Affiliations:** 1 Zayed University, Abu Dhabi, United Arab Emirates; 2 Kuban State Agrarian University, Krasnodar, Russia; 3 Engineering School, Viterbo, Italy

**Keywords:** Fractional differential equations, Novel coronavirus, Riesz wavelet system, Smoothed pseudosplines, Mathematical model

## Abstract

The well-known novel virus (COVID-19) is a new strain of coronavirus family, declared by the World Health Organization (WHO) as a dangerous epidemic. More than 3.5 million positive cases and 250 thousand deaths (up to May 5, 2020) caused by COVID-19 and has affected more than 280 countries over the world. Therefore studying the prediction of this virus spreading in further attracts a major public attention. In the Arab Emirates (UAE), up to the same date, there are 14,730 positive cases and 137 deaths according to national authorities. In this work, we study a dynamical model based on the fractional derivatives of nonlinear equations that describe the outbreak of COVID-19 according to the available infection data announced and approved by the national committee in the press. We simulate the available total cases reported based on Riesz wavelets generated by some refinable functions, namely the smoothed pseudosplines of types I and II with high vanishing moments. Based on these data, we also consider the formulation of the pandemic model using the Caputo fractional derivative. Then we numerically solve the nonlinear system that describes the dynamics of COVID-19 with given resources based on the collocation Riesz wavelet system constructed. We present graphical illustrations of the numerical solutions with parameters of the model handled under different situations. We anticipate that these results will contribute to the ongoing research to reduce the spreading of the virus and infection cases.

## Introduction

In March 2020, WHO has announced the novel coronavirus as a pandemic after the outbreak on the end of January 2020, when it was declared a public health emergency for the global. Since then, the pandemic has affected almost all countries around the world and killed more than 290,000 of people worldwide. The virus can easily spread from one to another, and no treatment or vaccine can do the needs [1, 2]. Even though a vaccine could be more than a year away, doctors are experimenting with drugs and therapies to help ease the virus symptoms/spreading.

Given the dangerous situation, many researchers started working on formulating models that best describe the dynamics of all possible parameters responsible for the daily cases reported including deaths, control the fatality rate, and prediction of COVID-19 behavior in future within a specific region. For example, Alberto [3] has developed a mathematical model to identify the number of days students could attend school to allow them a better learning experience while mitigating infections of COVID-19.

It is known that several models can describe a specific system, which is a challenging step. However, in this paper, we use the well-known parsimony principle, where the model should be constructed in a simple way as possible but also with complexity when needed.

Fractional derivatives have been proven to be a useful tool in a wide area of applications in science and engineering [4–15], including customary in groundwater analysis, the modeling of infection disease, and epidemic systems to discover and predict the spreading of many diseases. It is known that Covid-19 originated in bats and infected humans and can infect several animals such as cats and ferret. There is no case (up to date) of direct transmission from a bat to human, but yet a proposal says that there is a host-reservoir most likely involved between them. We consider the model presented in [16] developed based on

$$ Bats \quad \Rightarrow\quad Hosts\quad \Rightarrow\quad Reservoir\quad \Rightarrow\quad People $$

formulation setting in terms of the Atangana–Baleanu fractional derivative. In this paper, we use the Caputo fractional derivative definition to study the model. The advantage of using this definition is that it allows traditional and various types of ICs in creation of a dynamical model. Wavelets appear in a variety of advanced applications such as filter bank constructions arising in image processing. This is largely due to the fact that wavelets have the right structure to capture the sparsity in “physical” images, perfect mathematical properties such as its multiscale structure, sparsity, smoothness, compact support, and high vanish moments. It has many applications in fractional integral and differential equations (see, e.g., [17–30]).

Riesz wavelets in $L_{2}(\mathbb{R})$ have been extensively used in the context of both pure and numerical analysis in many applications due to their well prevailing and recognized theory and their natural properties such as sparsity and stability, which lead to a well-conditioned scheme. In this paper, we present an effective and accurate technique based on Riesz wavelets for solving the transmission model of COVID-19 based on the Caputo fractional derivative. The advantage of such wavelets lies on their simple structure in the reduced systems and in the powerfulness of obtaining approximated solutions for equations that have weakly singular kernels. The proposed method shows a good performance and high accuracy orders.

Let us recall some definitions and notation. A function $\phi \in L_{2}(\mathbb{R})$ is called refinable if

1.1 $$ \phi =\sum_{k\mathbb{Z}}{a[k]\phi (2\cdot -k)}, $$

where $a[k]\in \ell _{2}(\mathbb{Z})$ is finitely supported sequence, called the refinement mask of *ϕ*. The corresponding wavelet function is defined by

1.2 $$ \psi =\sum_{k\mathbb{Z}}{b[k]\phi (2\cdot -k)}, $$

where $b[k]\in \ell _{2}(\mathbb{Z})$ is finitely supported sequence, called the high pass filter of *ψ*.

In this paper, for $f\in L_{1}( \mathbb{R})$ (which can be extended to $L_{2}(\mathbb{R})$), we use the Fourier transform

$$ \hat{f}(\xi )=\frac{1}{\sqrt{2\pi }} \int _{\mathbb{R}}{e^{-ix\xi }f(x)\,dx.} $$

The Fourier series of the sequence *a* is defined as

1.3 $$ \hat{a}(\xi )=\sum_{k\in \mathbb{Z}}{a[k]e^{-ik\xi }}, \quad \xi \in \mathbb{R}. $$

Pseudosplines have attracted many researchers due to their significant contribution to both numerical computations and analysis. The constructions of pseudosplines track back to the well-known work by Daubechies et al. [31, 32], It is a family of refinable functions with compact support and has extensive flexibility in wavelets and applications. Pseudosplines are known as a generalization of many well-known refinable functions such as B-splines, interpolated, and orthonormal refinable functions [33]. We refer the reader to [31, 32, 34–38] and references therein for more detail.

## Riesz wavelets via smoothed pseudosplines

We use the smoothed pseudosplines introduced in [38] to construct Riesz wavelets and use them to apply our numerical scheme for solving different types of FIDEs. Pseudosplines of order $(p,q)$ of types I and II, $_{k}\phi _{(p,q)}$, $k=1,2$, are defined in terms of their refinement masks, where

$$ \bigl\vert {}_{1} \hat{a}_{(p,q)}(\xi ) \bigr\vert ^{2}=\sum_{m=0}^{q}{ \binom{p+q}{m} \bigl( \cos (\xi /2) \bigr)^{2(p+q-m)}\sin ^{2m}(\xi /2)}, $$

and

$$ {}_{2} \hat{a}_{(p,q)}(\xi )= \bigl\vert {}_{1} \hat{a}_{(p,q)}(\xi ) \bigr\vert ^{2}. $$

Note that the refinement mask of the pseudosplines of type I of order $(p,q)$ is obtained using the Fejér–Riesz theorem. The refinable pseudospline function generated using the above refinement masks is defined by

2.1 $$ _{k}\hat{\phi }_{(p,q)}(\cdot )=\prod ^{\infty }_{m=1}{_{k} \hat{a}_{(p,q)} \bigl( \cdot /2^{m}\bigr)},\quad k=1,2. $$

They are two types of smoothed pseudosplines defined by its refinable masks. For $r\geq p$, we have the smoothed refinable pseudosplines of type I ($k=1$) and II ($k=2$) of order $(r,p,q)$ such that

2.2 $$ _{k}\phi _{(r,p,q)}(\cdot )={}_{k}\phi _{p,q}\ast \chi ^{r-p}_{[- \frac{1}{2},\frac{1}{2}]}(\cdot ),\quad k=1,2, $$

where

$$ \chi ^{r-p}_{[-\frac{1}{2},\frac{1}{2}]}(\cdot )=\chi _{[-\frac{1}{2}, \frac{1}{2}]}\ast \cdots \ast \chi _{[-\frac{1}{2},\frac{1}{2}]}, \quad \text{for $(r-p)$-times}, $$

where $\chi _{A}$ is the indicator function of a set *A*. Similarly, the refinement masks of both types of $_{k}\phi _{(r,p,q)}$ for $k=1,2$, respectively, are given by

$$ \bigl\vert {}_{1} \hat{a}_{(r,p,q)}(\xi ) \bigr\vert ^{2}=\sum_{m=0}^{q}{ \binom{p+q}{m} \bigl(\cos (\xi /2) \bigr)^{2(r+q-m)}\sin ^{2m}(\xi /2)} $$

and, for $r\geq 2p$,

$$ {}_{2} \hat{a}_{(r,p,q)}(\xi )=\sum _{m=0}^{q}\binom{p+q}{m} \bigl( \cos (\xi /2) \bigr)^{2q+r-p}\sin ^{2m}(\xi /2). $$

Riesz wavelets have been extensively studied in the literature; see, for example, [39] and other references.

### Definition 2.1

We say that the set $\mathcal{M}(\psi ^{\ell })= \{ \psi ^{\ell }_{j,k}=2^{j/2}\psi ^{ \ell }(2^{j}\cdot -k),\ell =1,\dots ,N \} $, $\psi ^{\ell }\in L_{2}( \mathbb{\mathbb{R}})$, generates a Riesz wavelet in $L_{2}(\mathbb{R})$ if for any finitely supported sequence $\{ n^{\ell }_{j,k},\ell =1,\dots ,N; j, k\in \mathbb{Z} \} $, there exist positive numbers *c* and *C* such that

2.3 $$ c \sum_{\ell =1}^{N}\sum _{j\in \mathbb{Z}}\sum_{k\in \mathbb{Z}} \bigl\vert n^{ \ell }_{j,k} \bigr\vert ^{2}\leq \Biggl\Vert \sum_{\ell =1}^{N} \sum _{j\in \mathbb{Z}}\sum_{k\in \mathbb{Z}}n^{\ell }_{j,k} \psi ^{\ell }_{j,k} \Biggr\Vert ^{2} \leq C \sum_{\ell =1}^{N}\sum _{j\in \mathbb{Z}}\sum_{k \in \mathbb{Z}} \bigl\vert n^{\ell }_{j,k} \bigr\vert ^{2},\quad \forall g\in L_{2}(\mathbb{R}), $$

where

$$ \Vert g \Vert ^{2}= \langle g,g \rangle , \quad \text{and}\quad \langle f,g \rangle = \int _{\mathbb{R}}{f(x) \overline{g(x)}}\,dx. $$

If $\mathcal{M}$ in Definition 2.1 is a Riesz wavelet for $L_{2}(\mathbb{R})$, then we have the following expansion for any function $f\in L_{2}(\mathbb{R})$:

2.4 $$ f=\sum_{\ell =1}^{N}\sum _{j,k\in \mathbb{Z}}{ \bigl\langle f,\psi ^{ \ell }_{j,k} \bigr\rangle \psi ^{\ell }_{j,k}}. $$

Equation (2.4) can be truncated by

2.5 $$ \mathcal{V}_{M}f=\sum_{\ell =1}^{N} \sum_{j\leq M-1}\sum_{k\in \mathbb{Z}}{ \bigl\langle f,\psi ^{\ell }_{j,k} \bigr\rangle \psi ^{ \ell }_{j,k}}. $$

Let us provide some examples of Riesz wavelet systems. Note that *ϕ̂* is only implicitly known as an infinite product. We strongly recommend the reader to have a look at Han’s book [39] (p. 68, Sect. 6) to get a complete picture about how to plot these wavelets and details.

### Example 2.1

For $(r,p,q)=(6,2,1)$, we have the following refinable masks:

$$\begin{aligned}& {}_{1}\hat{a}_{(6,2,1)}(\xi ) = \frac{1}{2} ( \sqrt{3}+1 ) e^{- \frac{1}{2} 5 i \xi } \bigl(1+ (\sqrt{3}-2 ) e^{i \xi } \bigr) \cos ^{3} \biggl(\frac{\xi }{2} \biggr), \\& {}_{1} \hat{b}_{(6,2,1)}(\xi ) = e^{-i\xi } \overline{{}_{1}\hat{a}_{(6,2,1)}(\xi +\pi )}, \end{aligned}$$

where

$$\begin{aligned}& {}_{1}\phi _{(6,2,1)}(2 \cdot ) = {}_{1} \hat{a}_{(6,2,1)}(\cdot )\,{}_{1} \phi _{(6,2,1)}(\cdot ), \\& {}_{1}\psi _{(6,2,1)}(2 \cdot ) = {}_{1} \hat{b}_{(6,2,1)}(\cdot )\,{}_{1} \phi _{(6,2,1)}(\cdot ). \end{aligned}$$

Then $\mathcal{M}( {}_{1}\psi _{(6,2,1)})$ forms a Riesz wavelet system for $L_{2}(\mathbb{R})$. Note that the vanishing moment for the system is 6.

### Example 2.2

For $(r,p,q)=(9,3,2)$, we have the following refinable masks:

$$\begin{aligned}& {}_{2}\hat{a}_{(9,3,2)}(\xi ) = \frac{1}{4} \cos ^{10} \biggl( \frac{\xi }{2} \biggr) \bigl(-156 \cos (\xi )+33 \cos (2 \xi )+127\bigr), \\& {}_{2}\hat{b}_{(9,3,2)}(\xi ) = e^{-i\xi } \overline{{}_{2}\hat{a}_{(9,3,2)}(\xi +\pi )}, \end{aligned}$$

where ${}_{1}\hat{a}_{(9,3,2)}(\xi )$ is obtained using the Fejér–Riesz factorization theorem, so

$$ \bigl\vert {}_{1} \hat{a}_{(9,3,2)}(\xi ) \bigr\vert ^{2} \approx {}_{2}\hat{a}_{(9,3,2)}( \xi ), $$

where

$$\begin{aligned}& {}_{2}\phi _{(9,3,2)}(2\cdot ) = {}_{1} \hat{a}_{(9,3,2)}(\cdot )\, {}_{1} \phi _{(9,3,2)}(\cdot ), \\& {}_{2}\psi _{(9,3,2)}(2\cdot ) = {}_{1} \hat{b}_{(9,3,2)}(\cdot )\, {}_{1} \phi _{(9,3,2)}(\cdot ). \end{aligned}$$

**Figure 1:**
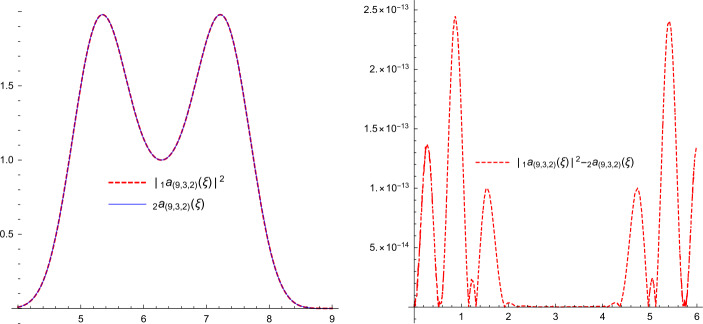
The graphs of the masks $\vert {}_{1} \hat{a}_{(9,3,2)}(\xi ) \vert ^{2}$ and $2\hat{a}_{(9,3,2)}(\xi )$ and their difference in Example 2.2

Then $\mathcal{M}( {}_{2}\psi _{(9,3,2)})$ forms a Riesz wavelet system for $L_{2}(\mathbb{R})$. Here we find ${}_{1}\hat{a}_{(9,3,2)}(\xi )$ numerically; see Fig. 1. Note that

$$ \bigl\vert \bigl\vert {}_{1} \hat{a}_{(9,3,2)}(\xi ) \bigr\vert ^{2}- {}_{2}\hat{a}_{(9,3,2)}( \xi ) \bigr\vert \leq \mathcal{O}\bigl(10^{-13}\bigr). $$

### Definition 2.2

For a real function $u(t)$ where $t,\alpha >0$, and $n\in \mathbb{N}$, we define the following known fractional derivative and integral operator:

- Caputo’s fractional derivative CFD,
  $$ \mathcal{D}^{\alpha }_{*}u(t)= \frac{1}{\Gamma (n-\alpha )} \int ^{t}_{0}{ \frac{u^{(n)}(x)}{(t-x)^{\alpha +1-n}}\,dx}, \quad n-1< \alpha \leq n. $$
- Riemann–Liouville fractional integral operator (R-LFI),
  $$ \mathcal{I}^{\alpha }u(t)= \frac{1}{\Gamma (\alpha )} \int ^{t}_{0}{ \frac{u(x)}{(t-x)^{1-\alpha }}\,dx},\quad n-1< \alpha \leq n. $$

## Transmission model and numerical algorithm based on Riesz wavelet fitting

**Figure 2:**
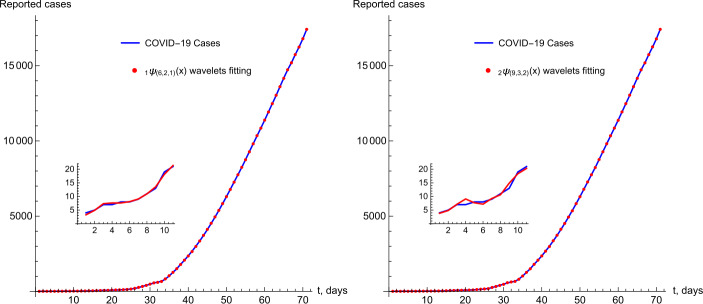
Reported cases of COVID-19 in the UAE with the Riesz wavelet fitting based on Examples 2.1 and 2.2

The original data are fitted by a set of discrete Riesz wavelet coefficients, where features can be extracted from these coefficients. The simulated data based on the Riesz wavelet systems are illustrated in Fig. 2.

We consider the following new modified transmission model obtained by changing the left-hand side of the system presented in [16] by changing the operator

$$ {}^{ABC}_{a} \mathcal{D}_{t}^{\alpha }u(t) \quad \mbox{to}\quad \mathcal{D}^{ \alpha }_{t}u(t) $$

for all unknown functions $S_{p}$, $E_{p}$, $I_{p}$, $A_{p}$, $R_{p}$, *M*, where *ρ* represents the fractional-order parameter. Note that the system is subject to nonnegative initial conditions. So the new system is defined as follows:

3.1 $$\begin{aligned}& \mathcal{D}^{\alpha }_{t}S_{p}(t) = \Pi _{p}-\mu _{p} S_{p}- \frac{\eta _{p}S_{p}(I_{p}+\psi A_{p})}{N_{p}}- \eta _{w}S_{p}M, \end{aligned}$$

3.2 $$\begin{aligned}& \mathcal{D}^{\alpha }_{t}E_{p}(t) = \frac{\eta _{p}S_{p}(I_{p}+\psi _{p} A_{p})}{N_{p}}+\eta _{w}S_{p}M-(1- \theta _{p})\omega _{p}E_{p}-\theta _{p}\alpha , \end{aligned}$$

3.3 $$\begin{aligned}& \mathcal{D}^{\alpha }_{t}I_{p}(t) = (1-\theta _{p})\omega _{p}E_{p}-( \tau _{p}+\mu _{p}) I_{p} , \end{aligned}$$

3.4 $$\begin{aligned}& \mathcal{D}^{\alpha }_{t}A_{p}(t) = \theta _{p}\rho _{p}E_{p}-(\tau _{ap}+ \mu _{p})A_{p}, \end{aligned}$$

3.5 $$\begin{aligned}& \mathcal{D}^{\alpha }_{t}R_{p}(t) = \tau _{p}I_{p}+ \tau _{ap}A_{p}- \mu _{p}R_{p}, \end{aligned}$$

3.6 $$\begin{aligned}& \mathcal{D}^{\alpha }_{t}M(t) = \varrho _{p} I_{p}+ \varpi _{p}A_{p}- \pi M. \end{aligned}$$

Given the model parameters and its values in Table 1, it is reasonable to consider this model with proper changes as it is formulated based on the resources and cases detected in Wuhan, China. Since the cases are spread to more than 280 countries around the world, some parameter values will be considered in the current study. For more detail about the formulation and stability results, we refer to [16]. To illustrate the fitting, we use some examples of Riesz wavelet systems to be used in the data fitting using the discrete Riesz wavelet transform defined in (1.3) based on different types of smoothed pseudosplines of orders I and II.

**Table 1:** Parameters description and estimated fitted values given $\rho =0.9$

Parameter	Description	Parameter value
$S_{p}(t)$	The susceptible cases	–
$E_{p}(t)$	The exposed cases	–
$I_{p}(t)$	The infected cases	–
$A_{p}(t)$	The asymptotically cases	–
$R_{p}(t)$	The recovered cases	–
*M*(*t*)	The infected cases	–
$\Pi _{p}$	Birth rate	341.706
$\mu _{p}$	Natural mortality rate	0.0000353513
$\eta _{p}$	Contact rate	0.01
$\psi _{p}$	Transmissibility multiple	0.01
$\eta _{w}$	Disease transmission coefficient	0.000001
$\theta _{p}$	The proportion of asymptomatic infection	0.09
$\omega _{p}$	Incubation period (bats)	0.00039
$\rho _{p}$	Incubation period (hosts)	0.001
$\tau _{p}$	recovery rate of $I_{p}$	0.1593
$\tau _{ab}$	recovery rate of $A_{p}$	0.95
$\varrho _{p}$	Contribution of the virus to *M* by $I_{p}$	0.0001
$\varpi _{p}$	Contribution of the virus to *M* by $A_{p}$	0.00089
*π*	Removing rate of virus from *M*	0.009

We provide a numerical algorithm based on the collocation method by discretizing the domain function across the Riesz wavelet system used to solve the model. The system is generated using the smoothed pseudosplines of types I and II with different orders. The model defined in Equations (3.1)–(3.5) can be reduced as follows:

$$\begin{aligned}& \frac{1}{\Gamma {(1-\alpha )}} \int _{0}^{t}{ \frac{S'_{p}(x)}{(t-x)^{\alpha }}\,dx} = \Pi _{p}-\mu _{p} S_{p}- \frac{\eta _{p}S_{p}(I_{p}+\psi A_{p})}{N_{p}}-\eta _{w}S_{p}M, \\& \frac{1}{\Gamma {(1-\alpha )}} \int _{0}^{t}{ \frac{E'_{p}(x)}{(t-x)^{\alpha }}\,dx} = \frac{\eta _{p}S_{p}(I_{p}+\psi A_{p})}{N_{p}}+\eta _{w}S_{p}M-(1- \theta _{p})\omega _{p}E_{p}-\theta _{p}\rho _{p}E_{p}, \\& \frac{1}{\Gamma {(1-\alpha )}} \int _{0}^{t}{ \frac{I'_{p}(x)}{(t-x)^{\alpha }}\,dx} = (1-\theta _{p})\omega _{p}E_{p}-( \tau _{p}+\mu _{p}) I_{p}, \\& \frac{1}{\Gamma {(1-\alpha )}} \int _{0}^{t}{ \frac{A'_{p}(x)}{(t-x)^{\alpha }}\,dx} = \theta _{p}\rho _{p}E_{p}-( \tau _{ap}+\mu _{p})A_{p} , \\& \frac{1}{\Gamma {(1-\alpha )}} \int _{0}^{t}{ \frac{R'_{p}(x)}{(t-x)^{\alpha }}\,dx} = \tau _{p}I_{p}+ \tau _{ap}A_{p}- \mu _{p}R_{p}, \\& \frac{1}{\Gamma {(1-\alpha )}} \int _{0}^{t}{ \frac{M'(x)}{(t-x)^{\alpha }}\,dx} = \varrho _{p} I_{p}+ \varpi _{p}A_{p}- \pi M. \end{aligned}$$

Using collocation method based on the nodes $t_{i}$, $i\in \mathbb{ N}$, in these equations, we obtain the following equations that generate a system of nonlinear equations to be solved numerically:

$$\begin{aligned}& -\mathcal{D}^{\alpha }_{t}S_{p}(t_{i})+ \Pi _{p}-\mu _{p} S_{p}(t_{i}) - \frac{\eta _{p}S_{p}(t_{i})(I_{p}(t_{i})+\psi A_{p}(t_{i}))}{N_{p}}- \eta _{w}S_{p}(t_{i})M(t_{i}) =0, \\& -\mathcal{D}^{\alpha }_{t}E_{p}(t_{i})+ \frac{\eta _{p}S_{p}(t_{i})(I_{p}(t_{i})+\psi A_{p}(t_{i}))}{N_{p}} \\& \quad {}+ \eta _{w}S_{p}(t_{i})M(t_{i})-(1- \theta _{p})\omega _{p}E_{p}(t_{i})- \theta _{p}\rho _{p}E_{p}(t_{i}) = 0, \\& - \mathcal{D}^{\alpha }_{t}I_{p}(t_{i}) + (1-\theta _{p})\omega _{p}E_{p}(t_{i})-( \tau _{p}+\mu _{p}) I_{p}(t_{i}) = 0, \\& -\mathcal{D}^{\alpha }_{t}A_{p}(t_{i}) + \theta _{p}\rho _{p}E_{p}(t_{i})-( \tau _{ap}+\mu _{p})A_{p}(t_{i}) = 0, \\& -\mathcal{D}^{\alpha }_{t}R_{p}(t_{i}) + \tau _{p}I_{p}(t_{i})+ \tau _{ap}A_{p}(t_{i})- \mu _{p}R_{p}(t_{i})= 0, \\& -\mathcal{D}^{\alpha }_{t}M(t_{i}) + \varrho _{p} I_{p}(t_{i})+ \varpi _{p}A_{p}(t_{i})-\pi M(t_{i})= 0. \end{aligned}$$

The parameter values listed in Table 1 were estimated based on some known results and assumptions, taking into consideration the effect of each subgroup/population in the virus spread. Based on official data on the COVID-19 in the UAE among the residents, we consider the estimation of the parameters of the dynamics of the virus. This due to the fact that the dynamics of the virus transmission from a country (e.g., the case study UAE) to another (China) does not change much. In addition, the other parameters are related to the structure of populations and has no affect on the nature of the virus.

The total population $N(0)$ of the UAE in 2019 is approximately 9.666 millions. The life expectancy in the UAE for the year of 2019 is 77.5. Therefore the natural mortality rate is $1/(77.5 \times 365)$. The birth rate is estimated by multiplying the value of total population times the mortality rate, so it is estimated by the value 341.706. For the initial values of the model, we consider the population size 9.666 millions for $t=0$. We assume that the number of infected people was 300 and, initially, there were no recovered cases, $R_{p}(0)=0$. Hence we have the following ICs:

$$ S_{p}(0)=\frac{9\text{,}344\text{,}440}{N_{p}(0)},\qquad A_{p}(0)= \frac{200}{N_{p}(0)}, \qquad E_{p}(0)= \frac{321\text{,}060}{N_{p}(0)}, \qquad M(0)= \frac{5000}{N_{p}(0)}. $$

**Figure 3:**
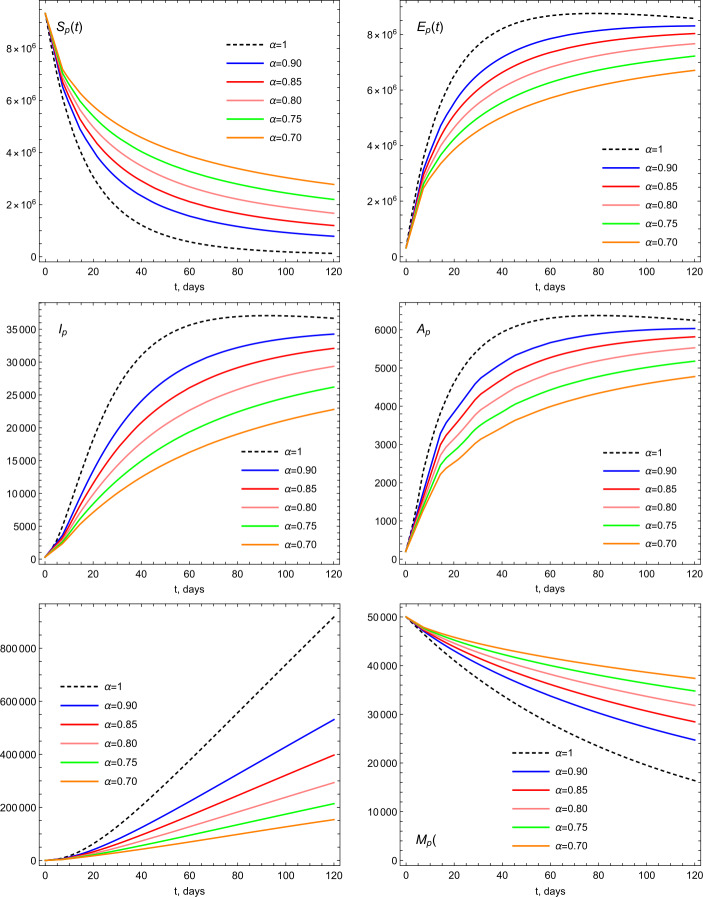
Illustrations of the dynamics of the model parameters using various values of *α*

**Figure 4:**
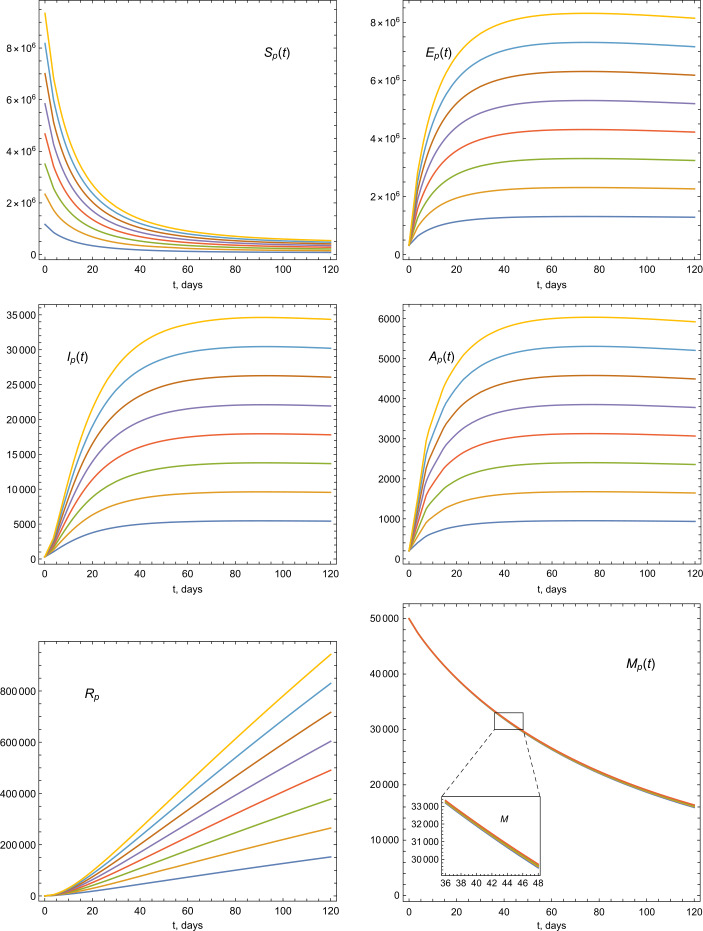
Illustrations of the dynamics of the model parameters using various ICs

**Figure 5:**
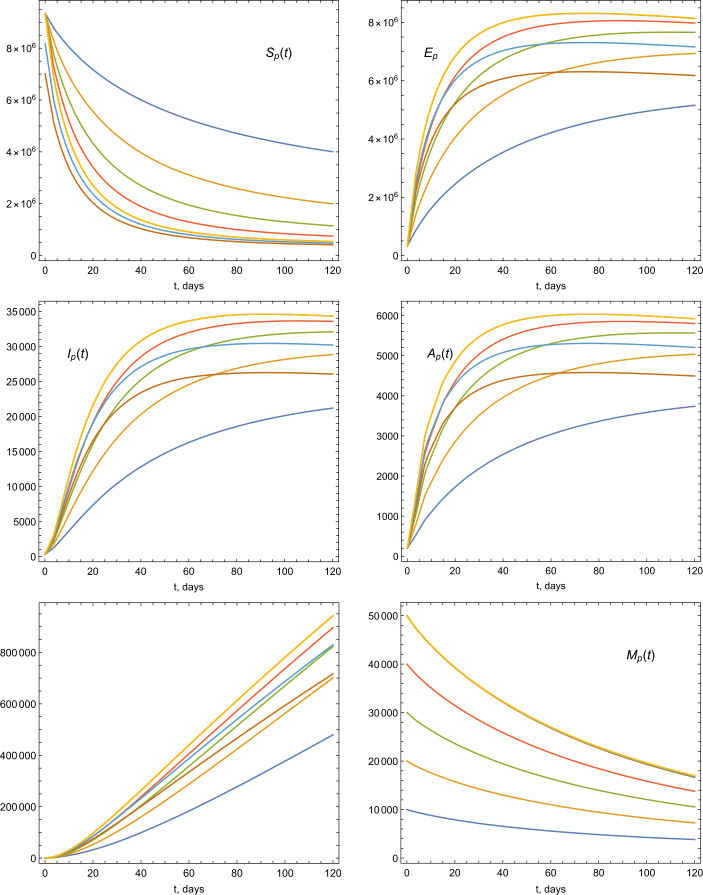
Illustrations of the dynamics of the model parameters using various ICs

**Figure 6:**
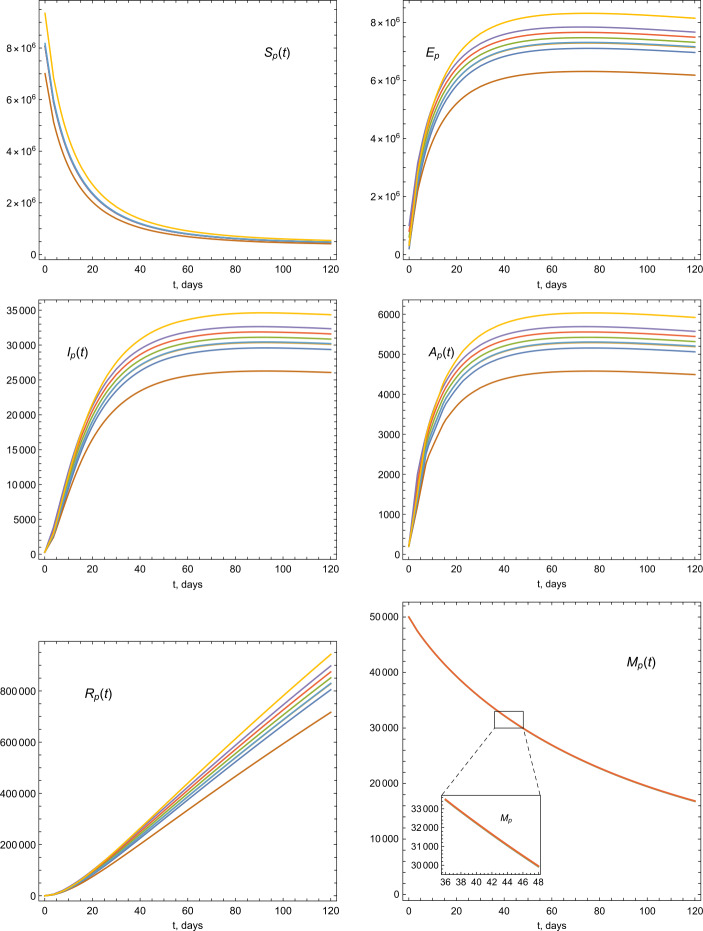
Illustrations of the dynamics of the model parameters using various ICs

Now we present some graphical illustrations based on the given parameter vales and simulation of the model given by Equations (3.1)–(3.5). The dynamics of COVID-19 based on different values of *α* is depicted in Fig. 3. In Figs. 4, 5, and 6, we provide illustrations of the stability of the model equilibrium by changing ICs, and we numerically calculate the parameters of the model by considering various ICs of $S_{p}$, *M*, and *E*.

## Conclusion

In this paper, we initially started the model formulation in terms of the classical integer-order derivative and then apply the Caputo fractional derivative. The model using the Caputo fractional derivative best describes the dynamics of the pandemic. The new resulting fractional model of COVID-19 describes the virus dynamics based on the resources and announced cases in the UAE.

We obtained some mathematical results for the model and simulated the original data and fitted it using a new family of Riesz wavelets based on refinable functions that have excellent properties such as symmetries and compact support. From the numerical simulation of the fractional model we notice that the fitted data were very accurate compared to the original data and may provide a good start to detect how the virus spreads.

We also provided graphical illustrations (Figs. 3–6) of the model parameters considering different values of fractional order *α* and various ICs. The presented figures describe the individuals behavior and its stability within the model equilibrium setting. It turns out that decreasing the order results in a decrease of the infection rates. We believe that, the suggested model is suitable to describe the dynamics of this virus and the nature of its spreading. We intended to consider the model with more data resources to better view the dynamics and virus spreading in the country. In future, we will extend the research to include various parameters and aspects, specifically, to predict the virus and positive cases under fitting the data with quarantine and stem cells factors.

In future, we are interested to work on the model presented in [3] by involving a new fractional-order derivative and specifically based on the UAE to further better recognize the dynamics of the new model to provide a feasible solution working successfully, especially for education authorities that are planning future activities.

## Data Availability

Not applicable.
